# Outcomes of Painful Hips After Hip Arthroscopy Surgery for Femoroacetabular Impingement

**DOI:** 10.7759/cureus.68190

**Published:** 2024-08-30

**Authors:** Gopalkrishna G Verma, Poornanand Goru, Rachael Heaton, Tarig Ahmed, Mobeen Ismail, Sanat V Shah

**Affiliations:** 1 Trauma and Orthopaedics, Manchester University NHS Foundation Trust, Manchester, GBR

**Keywords:** multidisciplinary approach, labral tear, preexisting chondral degeneration, individualized treatment plan, pain, postoperative, arthroscope, hip, impingement, femoroacetabular

## Abstract

The benefits of hip arthroscopic surgery for femoroacetabular impingement are well-established. Hip arthroscopic surgery rates have risen dramatically over the last decade. Some patients, however, may continue to experience hip symptoms after surgery and are dissatisfied with their inability to return to desired optimal activity levels. The purpose of the study is to understand the long-term outcomes of patients with painful hips after hip arthroscopy for femoroacetabular impingement. This is a retrospective study of the outcomes of painful hips after hip arthroscopy for femoroacetabular impingement, with four to 14-year follow-up from 2008 to 2022. A total of 84 hip arthroscopies were performed. Most of the patients had labral tear debridement and shaving of the aspherical femoral head also known as cam lesion, and five patients had repair for labral tear. There were eight patients who had bilateral hip involvement. There were 27 men and 57 females between the second to fifth decades. The electronic patient's records and radiological images were reviewed, and patient outcomes were graded as pain-free hip (asymptomatic) or painful hip (persistent pain and symptoms of instability). After hip arthroscopy surgery, 55% (46) of hips were graded pain-free in patients who were mostly in their 20s and 30s, while 45% (38) of hips had persistent pain. These patients were in their third or fifth decade. In the painful hip cohort, 33 patients had one hip arthroscopic surgery, while five patients had multiple repeat hip arthroscopies in the same hip over a three to six-year period. Bilateral hip arthroscopies were performed at different times in eight patients of which five individuals experienced painful hip outcomes. There were seven females and one male in their 30s and 40s. The labral tear was repaired in five patients, and two patients had painful hip outcomes. Both were females in their 20s and 30s. Patients with chronic painful hips after hip arthroscopic surgery were investigated to identify the cause of the pain. If no cause was established, then they were finally referred to pain specialist clinicians for pain management. This cohort had seven patients between 28 and 43 years. Six were female and one was male. Total hip replacement (THR) was performed in four patients (4.7%). Conversely, 95.3% of patients did not need THR during the study period of 14 years. Hip arthroscopy can be an effective treatment for femoroacetabular impingement. Careful patient selection and a holistic approach are vital for a good patient outcome. The success rate of the pain-free hip after hip arthroscopy decreases with increasing age of the patient, particularly in the female gender. Patients with grade II and more degenerative chondral changes do not perform well. Patients in their fourth and fifth decade can benefit from hip arthroscopy provided a comprehensive discussion of the expected outcomes is conducted prior to surgery. Overall, hip arthroscopy remains a valuable tool, but it is important to be conscious of its limitations and potential challenges.

## Introduction

Femoroacetabular impingement was first described by Ganz et al. in 1991 [1]. In this disease, there is morphological abnormality present, either within the head-neck junction of the femur with aspherical femur head, also known as cam impingement, or in the acetabular edge, which is pincer impingement. This leads to early pathologic contact during hip joint movements between the skeletal prominence of the femur and the acetabulum. Impingement can cause injury to the acetabular labrum or to the chondral surfaces of the acetabulum and femur head. This can manifest as a painful hip joint, a tear of the labrum, or degeneration of the cartilage of the hip joint [1]. Progressive degeneration of the cartilage develops into hip osteoarthritis [2].

Femoroacetabular impingement usually affects individuals between the second to fourth decades who are active and mostly engage in some form of sporting activity. There is a relationship between the intensity of activity and femoroacetabular impingement with an estimated incidence prevalence of 10%-15%. Approximately 14% of patients will present with isolated forms of cam or pincer impingement, whilst up to 86% will have a combination of both types of impingements [3]. Frank et al. reported impingement rates of cam at 37% and pincer at 67% in the systematic review [4].

Hip arthroscopic surgery for femoroacetabular impingement is a form of hip preservation procedure, which aims to ameliorate the pain and enhance functional performance. Early intervention before the onset of irreversible chondral degeneration can possibly delay the progressions of osteoarthritis and the need for total hip replacement (THR) [5,6]. Hip arthroscopy has well-established benefits, including lower complication rates, faster rehabilitation, and return to sports [7-10]. Hip arthroscopic surgery rates have risen dramatically over the last decade [7,11].

Multiple systemic reviews in the literature have condensed and complied with the current knowledge on the patient-reported outcome measures for hip arthroscopy for femoroacetabular impingement [6-8,12-14]. When performed in patients with appropriate indications, it can provide high satisfaction and low revision surgery rates [6,15]. Some patients, however, continue to experience painful hip symptoms after hip arthroscopy surgery. They are dissatisfied with their inability to return to their desired optimal activity levels [6]. Hip surgeons, general orthopaedic surgeons, and physiotherapists will be particularly interested in learning about the outcomes of painful hips following hip arthroscopy for femoracetabular impingement. This will allow them to provide specific and realistic expectations to the patients. The purpose of the study is to understand the long-term outcomes of patients with painful hips after hip arthroscopy for femoroacetabular impingement syndrome.

This article was presented as a meeting poster at the 2023 EFFORT annual scientific meeting on 24th May 2023.

## Materials and methods

This is a retrospective study from the single centre between 2008 and 2022. Patients of femoroacetabular impingement were identified from the reports of 303 patients who underwent magnetic resonance imaging arthrogram of the hips. A total of 84 hip arthroscopies were performed for femoroacetabular impingement. The minimum follow-up duration was four years, and the maximum is 14 years. There were 27 males and 57 females with ages ranging from 20 to 50 years. The right hip was symptomatic in 44 cases and the left hip in 40 cases.

The inclusion criteria to include the patient in the study were as follows: patients with symptoms, clinical findings, and radiological evidence of femoroacetabular impingement on magnetic resonance imaging hip arthrograms. Failure of non-operative treatment included physiotherapy, activity modifications, and anti-inflammatory medications. Patients were excluded if they had previous acetabular or femur fractures, Perthes disease, Ehlers-Danlos syndrome, or pigmented villonodular synovitis. In 79 patients, the labral tear was debrided along with shaving of the cam lesion, and five patients had repair for labral tear. There were eight patients who had bilateral hip arthroscopies at different time periods for symptoms in both hips.

The patient's medical notes and radiological images were reviewed from electronic patient records to determine the patient’s final outcome. A timeline of the patient's journey was charted from their initial visit to the orthopaedic clinic, followed by magnetic resonance imaging arthrogram of the hip, hip arthroscopy surgery, physiotherapy with rehabilitation, and subsequent follow-up visits in the outpatient clinics. The patient outcomes were graded as pain-free hip or painful hip at the end of the physiotherapy and rehabilitation programme. The pain-free hip outcome was concluded if the patient remained symptom-free at the time of discharge from the clinic, whilst patients with painful hip outcomes had persistent pain and the presence of instability symptoms in the hip. These patients were further investigated with repeat magnetic resonance imaging arthrograms of the hip to identify the cause of the pain.

All the hip arthroscopy procedures were performed by two senior surgeons. After the patient was anaesthetised, they were positioned supine on the hip arthroscopy traction table. Traction was applied to the operating side lower leg and foot to distract the hip joint by approximately 1 cm under image intensifier control. Conventional arthroscopy portals were used to approach the hip joints. Central compartment pathology was addressed initially, followed by decompression of the peripheral compartment. The patients were admitted as a day-case procedure. Physiotherapy was provided to the patient postoperatively as per the standard hip arthroscopy protocol. Patients were followed up in the outpatient clinic at six weeks after the surgery and subsequently at regular intervals of three to six months.

## Results

The outcomes of 84 hip arthroscopies were categorized into pain-free hips (55%, 46) and painful hips 45% (38). All the patients received physiotherapy after surgery. In 79 patients, the labral tear was debrided along with shaving of the cam lesion, and five patients had a repair for a labral tear. There were eight patients who had bilateral hip arthroscopies at different time periods for symptoms in both hips.

Most patients with pain-free hip outcomes were mostly in their 20s and 30s. The majority of patients had debridement of the labral tear, whilst three had labral repair. When these patients completed their physiotherapy and rehabilitation programme, they remained pain-free and were discharged. A cohort of patients with persistent painful hip outcomes (Table 1) were mostly in their third to fifth decade. Most of the patients had labral tears debrided and cam lesions shaved. Two patients had repair for their labral tear. Thirty-three of 38 patients had one hip arthroscopic surgery, whilst five patients had multiple repeat hip arthroscopies between two to three in the same hip over three to six years. Repeat hip arthroscopic surgery revealed either no labral tear despite radiological evidence, a small cam lesion that required further shaving, and, in one instance, a hypertrophied labrum that required additional debridement.

**Table 1:** Outcomes of painful hips after hip arthroscopy THR: Total hip replacement

	Age	Gender	Number of hip arthroscopy	Hip pathology
Painful hips 38 (45%) (33 + 4 + 1)				
	30-50 years	M & F	1	33 patients had debridement of labral tear
	24 years	F	2 (same hip)	2015, debridement of the labral tear was performed. 2018, additional debridement for recurrent labral tear
	36 years	F	2 (same hip)	2012, debridement of the labral tear was performed. 2015, additional debridement for recurrent labral tear
	37 years	F	2 (same hip)	2008, labral repair was performed. 2014, shaving of the hypertrophied labrum
	42 years	F	2 (same hip)	2012, labral repair was performed for chondrolabral junction tear along with debridement of femoral cam impingement. 2014, additional debridement of recurrent labral tear
	40 years	F	3 (same hip)	2011, debridement of the labral tear and shaving of femoral cam impingement was performed. 2012, the hip arthroscopy did not find a recurrence of tear in the labrum as was reported on repeat hip magnetic resonance imaging arthrogram. 2014, a second repeat hip magnetic resonance imaging for persistent painful hip demonstrated a recurrence of the labral tear which was debrided arthroscopically.
Acetabular labral tear (2 patients)				
	23 years	F	2 (same hip)	2009, initial labral repair was performed for chondrolabral junction tear. 2014, debridement of hypertrophied labrum was required.
	39 years	F	2 (same hip)	2012, labral repair was performed for chondrolabral junction tear. 2014, shaving of the minor femoral cam impingement.
Chronic hip pain referred to pain specialist (7 patients)				
	28 years	F	3 (same hip)	2011, debridement of the labral tear and shaving of the cam lesion. 2012, hip arthroscope did not demonstrate any recurrence of the tear as was suspicious on the magnetic resonance hip arthrogram. 2014, repeat hip arthroscopy to shave the minor residual cam lesion to address persistent hip pain
	35 years	F	1	2013, debrided the labral tear and shaving of the cam lesion.
	38 years	F	2 (same hip)	2012, debrided the labral tear and shaving of the cam lesion. 2015, repeat hip arthroscopy to shave the minor residual cam lesion
	40 years	F	2 (same hip)	2011, debrided the labral tear and shaving of the cam lesion. 2012, repeat hip arthroscopic shaving of the residual cam lesion.
	42 years	F	2 (left hip) 1 (right hip)	2012, Left hip arthroscopy repaired the chondrolabral junction tear with suture anchor. Cam lesion was shaved. 2014, repeat left hip arthroscopy stabilised the loose chondral flaps with arthroscopic wand and shaved the residual minor cam lesion. 2012, Right hip arthroscopy debrided the labral tear and shaving of the cam lesion.
	43 years	M	1	2017, debrided the labral tear and shaving of the cam lesion.
	45 years	F	1	2014, debrided the labral tear and shaving of the cam lesion. (This patient was previously treated by pain-team for persistent neck pain since 2009)
Total hip replacement (4.7%) (4 patients)	28 years & 50 years	F	1	Time duration between initial hip arthroscopy and THR was 4 and 6 years
	45 years & 52 years	M	1	Time duration between initial hip arthroscopy and THR was 3 and 12 years

Bilateral hip arthroscopies were performed in seven females and one male. These hip arthroscopes were performed over two to three years duration. Five individuals continued to have painful hips, whilst three patients had pain-free hip outcomes. Amongst these, five patients with painful hips, there were four females in their third decade and one male in his fourth decade. 

Two patients who underwent labral repair continue to experience painful symptomatic hips. Both are females in their 20s and 30s. The 23-year-old underwent initial labral repair. Magnetic resonance imaging arthrogram for painful hip six years later demonstrated a hypertrophied labrum, which required arthroscopic debridement. Another 39-year-old patient after initial labral repair required shaving of a minor cam lesion as demonstrated on magnetic resonance imaging hip arthrogram after two years.

Patients with chronic painful hips after hip arthroscopic surgery were further investigated to identify the cause of the pain. When no cause was determined then they were eventually referred to pain specialist clinicians for pain management. The majority were females in their third to fifth decades. Four patients had multiple repeat hip arthroscopes in the same hip for ongoing pain. One female patient was previously treated by the pain team for chronic neck pain.

Four patients required total hip replacement (THR) in their fourth and fifth decades. In all these patients, the preoperative magnetic resonance imaging arthrogram of the hip revealed degenerative chondral changes grades II-III as per the Outerbridge grading and during hip arthroscopy [16]. Conversely, 95.3% of patients did not need THR during the study period of four to 14 years.

## Discussion

In this study of 84 hip arthroscopies, most of the patients had labral debridement, rather than labral repair (94% vs 06%) [7,10,17,18]. Following hip arthroscopy for femoroacetabular impingement, the patients were followed up with a minimum of four years and a maximum of 14 years [7,8,12]. This probably allowed a reliable evaluation and insights into the long-term prognosis for pain-free and painful hip outcomes, risk factors for persistence of pain in the hip, and potential treatment alternatives for the patient who continues to experience pain after the surgery [12,13,19,20].

A significant proportion (55%) of the patients achieved pain-free hip outcomes after a single hip arthroscopy procedure, which suggests that the surgery is effective in addressing symptoms of femoroacetabular impingement in younger patients. Sogbein et al. in the systemic review, concluded that 72.2% of patients were satisfied and 22% had painful hip outcomes after hip arthroscopy [13]. Most of the patients had debridement of the labrum and cam lesion. Younger patients outperformed the older group.

Persistent painful symptomatic hips were the outcome in 45% of patients who were in their 30s and 50s. This highlights the need for careful patient selection and comprehensive discussion with the patient regarding their diagnosis and outcomes of hip arthroscopic surgery [20,21]. It is vital to methodically manage patient’s expectations after surgery in relation to multiple associated factors, which will influence the outcomes in the postoperative period. Age seems to be a factor, with a higher prevalence of painful hip outcomes in patients in their third, fourth, and fifth decades [7,8,13]. The female gender was more vulnerable to disappointing outcomes [8,13].

Preexisting chondral damage grades II-III in the hip joint correlated with painful hip outcomes that required total hip replacement after three to 12 years [7-9,13,14,19,21]. Huang et al. noted no conversion to total hip replacement in their study with a five-year follow-up for hip arthroscopy for femoroacetabular impingement [22]. Hwang et al. concluded total hip replacement conversion rates of 3.1% over 20.33 years of follow-up [17]. Modified Outerbridge grade IV cartilage damage was identified as a predictor of conversion to total hip replacement [12,16].

A multidisciplinary team approach is a must for patients with chronic painful hip [21,23] after hip arthroscopy before considering referral to a pain specialist for their pain management. A recommended multidisciplinary team should include orthopaedic surgeon, physiotherapist, pain physician, and patient. Additional multi-specialty clinicians and allied health professionals may form part of this multidisciplinary team depending on the patient’s comorbidities and background needs. Amongst the patients who were referred for pain management, female patients represented the majority. Patients who have previously been treated by a pain specialist for another musculoskeletal condition are more likely to experience painful hip outcomes following surgery. Repeat hip arthroscopies in painful hips do not ensure a successful pain-free result [8].

An individualized treatment plan can be formulated for each patient using a holistic approach, which takes into account all the multiple factors mentioned above, that may influence the patient outcomes in the postoperative period. This process encourages patients to engage actively in their treatment as most or all their needs are addressed. They are more likely to adhere to the recommended treatment plans, and possibly individualized treatment plans may assist effectively in managing postoperative wound pain. Positive experience with their treatment will help in smoother and quicker recovery and possibly foster a better outcome for the patient [24].

The study has few limitations as it did not include body mass index (BMI) or other radiological characteristics (acetabular index, lateral edge angle, hip offset, femoral anteversion) that are known to influence outcomes. We recommend incorporating these in future studies.

## Conclusions

Hip arthroscopy can be an effective and beneficial treatment for femoroacetabular impingement. Careful patient selection is crucial. The success rate of pain-free hip outcomes after hip arthroscopy decreases with increasing age of the patient, particularly in the female gender. Patients with degenerative chondral damage of grades II and above usually do not perform well. Patients in their fourth and fifth decade can benefit from hip arthroscopy provided that a comprehensive discussion of expected outcomes is conducted prior to surgery. Patients with previous or ongoing treatment with a pain specialist are more likely to have painful hip outcomes after hip arthroscopy surgery. Repeat hip arthroscopies in painful hips outcomes do not ensure a successful result. Hip arthroscopy surgery may not necessarily delay the need for total hip replacement. Overall, hip arthroscopy remains a valuable tool, but it is important to be conscious of its limitations and potential challenges.
